# Rapid Normalization of Amniotic Fluid Index Following Discontinuation of Olmesartan: A Case Report

**DOI:** 10.1055/a-2816-0125

**Published:** 2026-03-09

**Authors:** Annika Van Oosbree, Therese Larson, Hannah Conley, Matthew Bridges, Pedro Argoti, Giancarlo Mari

**Affiliations:** 1 Division of Maternal-Fetal Medicine, Medical University of South Carolina; Charleston, South Carolina, United States; 2 Department of Radiology, Medical University of South Carolina; Charleston, South Carolina, United States; 3 Division of Maternal-Fetal Medicine, Baylor University; Houston, Texas, United States

**Keywords:** olmesartan, angiotensin receptor blockers, ARBs, anhydramnios

## Abstract

**Background:**

Angiotensin II receptor blockers (ARBs) are commonly used for hypertension but are contraindicated in pregnancy due to risks of oligohydramnios, renal dysgenesis, and pulmonary hypoplasia from suppression of the fetal renin–angiotensin system. Olmesartan, a frequently prescribed ARB, has a longer receptor binding half-life and higher affinity than other ARBs, producing more potent and sustained antihypertensive effects. Emerging evidence suggests that stopping ARBs during pregnancy may allow recovery of amniotic fluid and renal function.

**Case:**

A 30-year-old primigravida with chronic hypertension presented at 24 weeks' gestation while taking olmesartan. Ultrasound revealed anhydramnios with a normal-appearing fetal genitourinary tract. Olmesartan was discontinued and replaced with labetalol. Within 2 weeks, the amniotic fluid index normalized, and subsequent ultrasounds showed sustained recovery. At 34 weeks, she delivered a viable male infant with reassuring renal function and only mild, improving calyceal dilation on postnatal ultrasound.

**Conclusion:**

This is, to our knowledge, the first reported case of reversible anhydramnios associated with first- and second-trimester olmesartan exposure. The favorable outcome highlights the potential for reversibility of ARB-related fetopathy with timely cessation. Clinicians should consider serial ultrasound monitoring before recommending termination, as early drug withdrawal may restore amniotic fluid and support normal neonatal outcomes.

## Introduction

Angiotensin II receptor blockers (ARBs) are first-line antihypertensive agents in the general population but are contraindicated in pregnancy due to the risk of fetal complications, including oligohydramnios, renal dysgenesis, and pulmonary hypoplasia. These effects result from suppression of the fetal renin–angiotensin system, reducing renal perfusion and urine output. 1 2 Olmesartan is a commonly-prescribed ARB that has been shown to have more robust effects on both systolic and diastolic blood pressures compared with other drugs in this class. 3 4 5 The American College of Obstetricians and Gynecologists recommends avoidance of ARBs in pregnancy due to the risk of fetal malformations and growth restriction. 6 Emerging evidence based on case reports and series demonstrates that discontinuation of ARBs in the first and second trimester of pregnancy may allow for recovery of amniotic fluid volume and renal function. We present a case of anhydramnios after first- and second-trimester olmesartan exposure that promptly resolved following drug cessation, resulting in a favorable neonatal outcome, highlighting the potential for reversibility of ARB-related fetopathy.

## Case Presentation

**Fig. 1:**
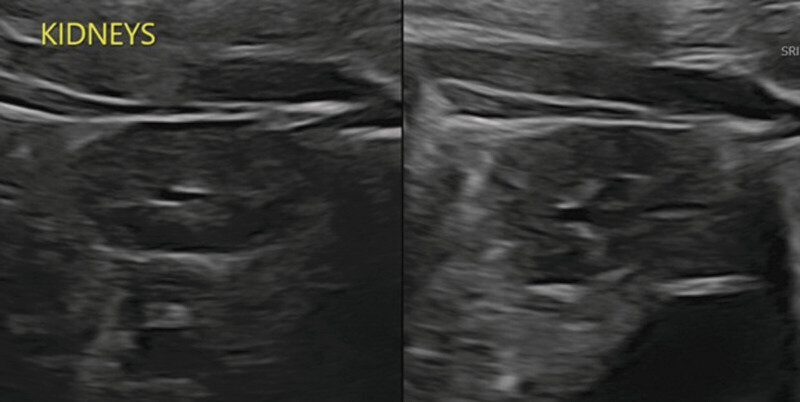
Normal-appearing fetal kidneys.

A 30-year-old G1 at 24 ^3/7^ weeks with chronic hypertension (CHTN) presented with severe-range blood pressures concerning for a CHTN exacerbation versus superimposed preeclampsia. She had been on olmesartan and triamterene/HCTZ prior to pregnancy recognition. She reported compliance with this medication regimen. Ultrasound showed anhydramnios with a normal-appearing fetal genitourinary system ( Fig. 1 ); rupture of membranes was ruled out with an amniotic fluid dye test. Intravenous antihypertensives were used acutely, and she was transitioned to labetalol 400 mg three times per day. She was ultimately diagnosed with CHTN exacerbation and discharged.

**Fig. 2:**
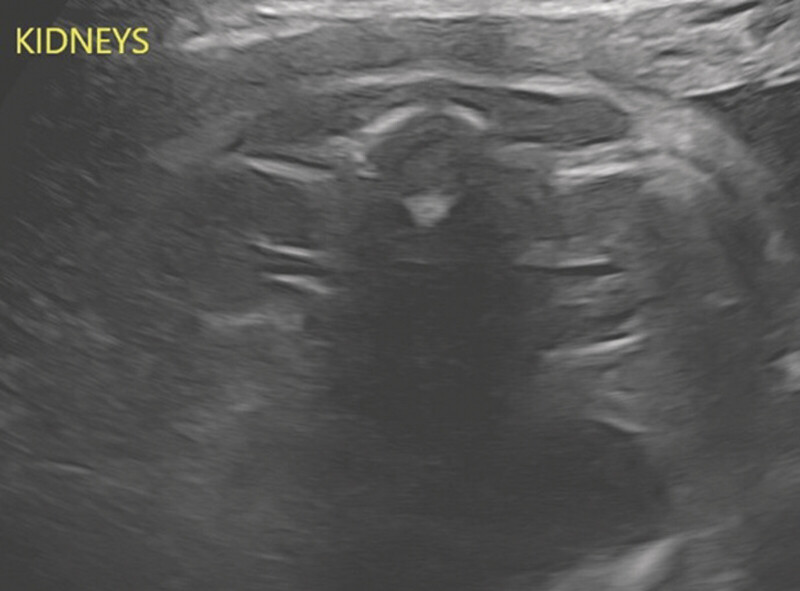
Amniotic fluid volume by gestational age.

She was monitored outpatient with serial ultrasounds. A week later, the ultrasound showed normal amniotic fluid volume. Two weeks after discontinuation, the amniotic fluid index (AFI) was improved to 14.0. Subsequent ultrasounds at 29 ^3/7^ weeks gestational age and 33 ^5/7^ weeks gestational age also showed improvement in AFI (20.6 and 14.0, respectively; see Table 1 and Fig. 2 ). Fetal anatomy was completed and was unremarkable.

**Table 1:** Ultrasounds throughout pregnancy

Gestational Age (wk)	BPD (mm)	HC (cm)	AC (cm)	FL (cm)	AFI (cm)	MVP (cm)	UA − S/D	Comments
25 ^4/7^	60.6	229.8	207.5	46.8	0	0	3.50	Bilateral kidneys are visualized
27 ^3/7^	NA	NA	NA	NA	14.8	4.9	NA	Bladder and stomach visualized
29 ^3/7^	73.8	277.7	249.9	56.3	20.6	6.1	NA	NA
33 ^5/7^	82.5	296.1	288.1	63.8	14.0	4.7	NA	NA

**Fig. 3:**
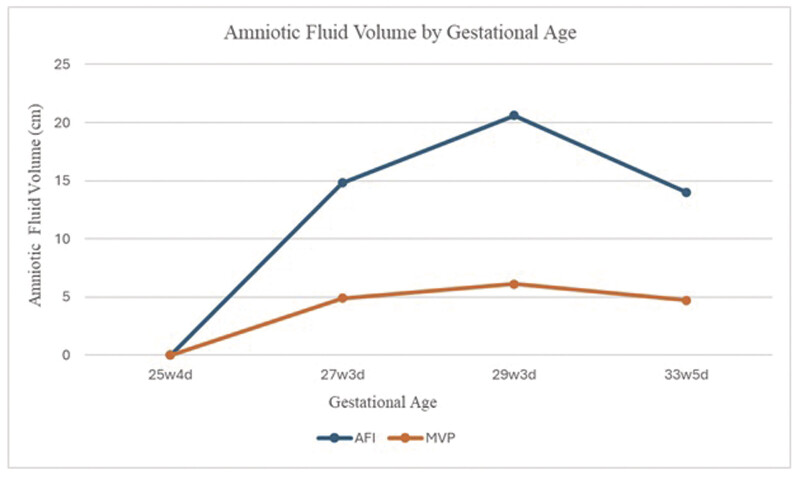
Renal ultrasound at 1 day old.

**Fig. 4:**
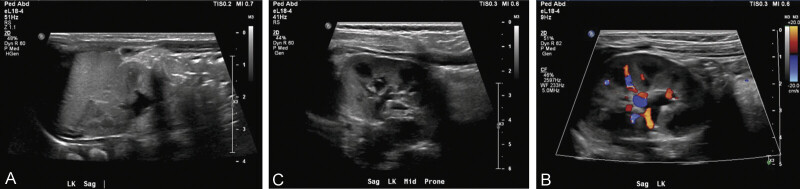
Renal ultrasound at approximately 6 months old.

At 33 ^4/7^ weeks of gestational age, she was diagnosed with superimposed preeclampsia with severe features. Induction of labor was initiated at 34 weeks, and she vaginally delivered a viable male infant weighing 2,390 g with APGARs of 6 and 7 at 1 and 5 minutes, respectively. His creatinine was 0.7 at birth and remained 0.7 to 0.8 throughout his admission. The infant's NICU course was complicated only by a renal ultrasound showing mild left-sided central calyceal dilation ( Fig. 3 ). He was ultimately discharged on day 17 of life. He is being followed up for the left-sided central calyceal dilation, which is improving ( Fig. 4 ).

## Discussion

Fetopathy related to renin–angiotensin system inhibitors, including angiotensin-converting enzyme inhibitors and ARBs, is well recognized, particularly with second- and third-trimester exposure. The classical findings include oligohydramnios, fetal growth restriction, calvarial hypoplasia, renal tubular dysgenesis, and pulmonary hypoplasia. 6 Renal abnormalities are frequently reported, most commonly renal tubular dysgenesis of the proximal tubule. Other findings include glomerular cysts, vascular thickening, and interstitial fibrosis, which may present on prenatal ultrasound as enlarged, hyperechogenic kidneys. 7 8 The pathophysiology is thought to arise from suppression of the fetal renin–angiotensin system, leading to impaired renal perfusion, reduced fetal urine output, and consequently, oligohydramnios development.

Although these complications have historically been associated with poor prognosis, several reports demonstrate reversibility after discontinuation of the offending agent. Chisholm et al described anhydramnios at 27 weeks in a patient on benazepril that normalized within 12 days. 9 Muller and James reported oligohydramnios with cranial abnormalities after benazepril exposure until 24 weeks, which resolved after withdrawal, though complicated by growth restriction. 10 Wei et al noted normalization of amniotic fluid within 8 days of stopping losartan at 32 weeks, with normal neonatal renal findings. 11 Similarly, Munk et al described improvement in amniotic fluid and postnatal resolution of renal changes following candesartan discontinuation. 12 Cases with good prognoses involving candesartan, telmisartan, losartan, and olmsartan 13 14 15 16 17 ; on the other hand, poor prognoses have been reported involving candesartan and valsartan 18 19 ( Table 2 ).

**Table 2:** Summary of ARB exposure cases

Reference	ARB used	Gestational age at presentation (or when oligohydramnios was diagnosed; wk)	Immediate fetal/neonatal outcome	Long-term outcome/follow-up
Briggs et al (2001) 18	Valsartan	24	Anhydramnios, pulmonary hypoplasia, very small placenta	IUFD
Pietrement et al (2003) 13	Telmisartan	33	Oligohydramnios	Neonatal survival. Acute renal failure without resolution on day 3 of life
Berkane et al (2004) 14	Valsartan	20	None	Neonatal survival
Bakkum et al (2006) 15	Losartan	27	Oligohydramnios resolved after cessation > inferior vena cava thrombosis	Neonatal survival
Bass and Faix (2006) 16	Losartan	29	Anhydramnios, empty fetal bladder, transient renal failure	Neonatal survival. Renal tubular acidosis
Simonetti et al (2006) 19	Candesartan	31 (delivered at that time)	Limb contractures, skull hypoplasia with microcephaly, underdeveloped valvarial bones, RDS, moderate oliguria	Neontal survival. Neurocognitive challenges (cognitive and linguistic), small hyperechogenic kidneys
Celetano et al (2008) 17	Olmesartan	29	Oligohydramnios and fetal renal impairment	Neonatal survival. No long-term effects
Munk et al (2010) 12	Candesartan	22	Severe oligohydramnios and fetal renal edema	Neonatal survival. Reversible fetal renal failure with recovery after cessation of candesartan

A larger review examined 83 fetuses exposed to ARBs in the second or third trimester, finding oligohydramnios in 58 cases. Of these, 19 demonstrated resolution following drug withdrawal, with outcomes ranging from perinatal death to complete recovery. Resolution generally occurred within 1 to 6 weeks, suggesting that the degree of injury may depend on both the timing and duration of exposure. 1

Olmesartan, like other ARBs, selectively antagonizes the angiotensin II type 1 (AT1) receptor, leading to vasodilation and reduced aldosterone secretion. However, it differs from many ARBs in its pharmacology and metabolism. It has one of the longest receptor binding half-lives among ARBs, leading to more sustained AT1 receptor blockade and potent, consistent blood pressure control. Olmesartan also shows higher receptor affinity compared with several other ARBs, which may explain its comparatively stronger antihypertensive effects at standard doses. 20 These properties make its pharmacodynamics somewhat distinct, although clinically it shares the class's same fetal risks. Both AT1 and AT2 receptors play a role in intrauterine development, though the AT2 receptor is predominant in fetal tissues, playing a role in organogenesis, vascular development, regulation of blood flow, and growth. 21 The AT1 receptor is more prevalent in the placenta, while the AT2 receptor is absent in some portions of the placenta. 22

The present case, to our knowledge, is the first-described case of olmesartan exposure during the first and second trimesters with discontinuation resulting in prompt, complete resolution of AFI. This is particularly significant given its higher AT1 receptor affinity than other ARBs. Not only did the AFI resolve, but the neonate's postnatal course was notable only for mild calyceal dilation, which improved on follow-up. Creatinine remained normal throughout his NICU course. This case highlights that early discontinuation of ARBs—specifically olmesartan, which is more potent than others—can lead to complete recovery of AFI and subsequent favorable neonatal outcomes. Given this case and the other previously-reported cases suggesting reversibility of anhydramnios in ARB exposure, we suggest recommending observation via serial ultrasounds rather than outright termination of pregnancy in the setting of first- and second-trimester ARB exposure.
